# Clinical Outcomes of Full-Thickness Macular Holes with Epiretinal Proliferation Without Posterior Vitreous Detachment

**DOI:** 10.3390/medicina61111975

**Published:** 2025-11-04

**Authors:** Kota Kakehashi, Reio Sekine, Tatsuya Jujo, Naoto Uchiyama, Akiko Endo, Naoto Tokuda, Yasushi Kitaoka

**Affiliations:** Department of Ophthalmology, St. Marianna University School of Medicine, 2-16-1 Sugao, Miyamae-ku, Kawasaki 216-8511, Kanagawa, Japan; kota.kakehashi@marianna-u.ac.jp (K.K.); t2jujo@marianna-u.ac.jp (T.J.); naoto.uchiyama@marianna-u.ac.jp (N.U.); akiko.endo@marianna-u.ac.jp (A.E.); tokunao@nifty.com (N.T.); kitaoka@marianna-u.ac.jp (Y.K.)

**Keywords:** full-thickness macular hole, epiretinal proliferation, epiretinal membrane, posterior vitreous detachment, lamellar macular hole

## Abstract

*Background and Objective*: To elucidate the clinical characteristics of full-thickness macular holes (FTMHs) with epiretinal proliferation (EP) without posterior vitreous detachment (PVD). *Material and Methods*: A retrospective and exploratory study reviewed clinical records of patients with FTMHs with EP without PVD (5 eyes: EP group) and FTMHs without EP without PVD (32 eyes: non-EP group). Swept-source OCT images were analyzed for macular structure. Statistical comparisons were made between clinical characteristics and surgical outcomes. *Results*: The EP group had two eyes with non-closure of macular holes (40%) (*p* = 0.01) and three eyes with ellipsoid zone defects (60%) (*p* = 0.01 compared with the non-EP group). *Conclusions*: Although limited by a small sample size, this pilot study suggests that the observed trends—such as a lower closure rate and more outer retinal disruption in the EP group—may indicate differences in pathogenesis and surgical outcomes compared to the non-EP group. However, these findings should be interpreted with caution due to statistical limitations. Further prospective studies with larger cohorts are needed to validate these preliminary findings and better understand the underlying pathogenesis.

## 1. Introduction

Recent developments in optical coherence tomography (OCT) have led to enhanced clarity of OCT images, enabling clinicians to evaluate the vitreomacular interface with greater precision and accuracy [1]. OCT scans reveal the presence of uniform material with moderate reflectivity surrounding both lamellar macular holes (LMHs) and full-thickness macular holes (FTMHs) in certain cases [2]. Epiretinal proliferation (EP) was first described as “thick” or “thicker” epiretinal membrane (ERM), and later renamed “lamellar macular hole-associated epiretinal proliferation” (LHEP) by Pang et al., as it was believed to be present only in association with LMHs [2]. However, subsequent reports showed that the presence of this epiretinal material is not limited to LMHs, as it can also be found in FTMHs, in the presence of posterior uveitis, and even associated with macular pucker [3]. Therefore, LHEP has been redefined by Hubschman et al. as EP [4].

Given the rarity of EP occurrence in eyes with FTMHs, limited research has explored its origin and composition within this context, despite the investigations conducted by several authors [3,5,6,7,8]. It has also been reported that stage-2 FTMHs, i.e., without posterior vitreous detachment (PVD), with EP are significantly less common than stage-4 ones, i.e., with PVD [7]. The aim of this study was to explore the potential clinical characteristics of FTMHs with EP in the absence of posterior vitreous detachment (PVD).

### 1.1. Patients and Methods

From June 2017 to March 2024, clinical records of consecutive patients showing evidence of FTMHs with EP without PVD or FTMHs without EP and without PVD were reviewed for this retrospective study. FTMHs were identified using the categorization system of the International Vitreomacular Traction Study Group [9]. FTMHs without EP and without PVD were classified based on the criteria established by Gass [10]. The swept-source OCT (SS-OCT) images were analyzed to verify the occurrence of a complete neurosensory retinal defect at the fovea. Patients with lamellar macular holes (LMHs) or macular hole (MH)-related retinal detachment were excluded. Furthermore, patients with a history of ocular trauma, previous vitreoretinal surgeries, or other retinal diseases were omitted.

Every participant underwent general ophthalmic analysis, consisting of binocular indirect ophthalmoscopy, fundus photography, slit-lamp biomicroscopy with a noncontact lens, and SS-OCT imaging. Clinical factors such as age, sex, axial length, and best-corrected visual acuity (BCVA) were documented. BCVA was measured using a Landolt C chart during the primary examination and again at 1, 3, and 6 months postoperatively. For statistical analysis, decimal BCVAs were converted into logarithm of the minimum angle of resolution (logMAR) units.

The presence of PVD at the optic disc or fovea was determined by reviewing SS-OCT images and confirmed during surgery. Comparisons were made between the clinical characteristics, preoperative and postoperative macular structures, macular hole closure rates, and BCVA in the FTMH with EP without PVD group (EP group) and the FTMH without EP and without PVD group (non-EP group).

### 1.2. Evaluations Using Swept-Source Optical Coherence Tomography

The macular region was imaged with a swept-source OCT (SS-OCT) device (Zeiss PLEX Elite 9000; Carl Zeiss Meditec Inc., Jena, Germany) with a 12 mm scan length, with the eye in the primary gaze position. Before surgery, the macular hole (MH) diameter was calculated by measuring the distance of the neurosensory retinal defects on the SS-OCT images. The average of the minimum and basal MH sizes was utilized for statistical analysis.

**Figure 1:**
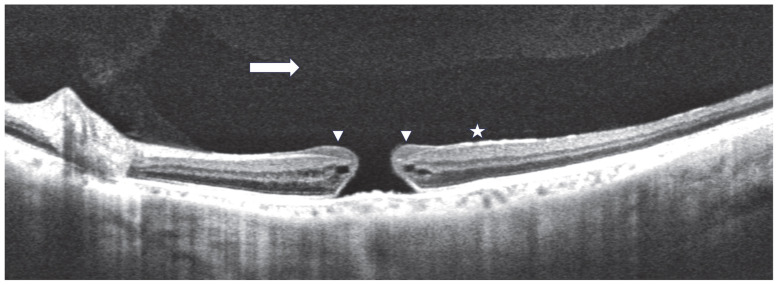
A full-thickness macular hole (FTMH) without posterior vitreous detachment (PVD) accompanied by epiretinal proliferation (EP). In swept-source OCT images, it is clear that there is no PVD or that vitreous pockets are easily observable (arrows). EP is present near the fovea, appearing as a thick, homogeneous, isoreflective preretinal material over the internal limiting membrane (arrowhead). The epiretinal membrane is visible as a highly reflective line on the internal limiting membrane (star). Scale bar = 100 μm.

EP was identified as a homogenous, isoreflective preretinal material located above the internal limiting membrane (ILM) (Figure 1) [4]. SS-OCT imaging was employed to distinguish EP from an ERM [11,12]. ERM was characterized as a hyperreflective layer applying traction on the inner retinal surface, often displaying retinal folds and sometimes featuring hyporeflective spaces between the ERM and ILM. The presence of EP and ERM was independently determined by two assessors (T.J. and R.S.), both blinded to the patients’ ophthalmologic findings. In cases where there was disagreement, a third investigator (Y.K.) reviewed the findings and led discussions to reach a final consensus. At 1, 3, and 6 months post-surgery, the presence of a defect in the ellipsoid zone (EZ) was also assessed.

### 1.3. Surgical Procedures

The surgical procedures were carried out by skilled vitreous surgeons under local anesthesia. Depending on the surgeon’s preference, 25-gauge or 27-gauge vitrectomy instruments were used. In every case, the core of the vitreous humor was excised, and the surgeons confirmed the presence of posterior hyaloid adhesions to the macula. The posterior hyaloid membrane was carefully peeled off using forceps or removed via suction with a vitreous cutter. The ILM was stained with Brilliant Blue G and peeled across the entire macular region. Subsequently, the vitreous cavity was filled with 25% sulfur hexafluoride (SF6), and patients were instructed to keep a facedown position for a period from one to several days.

### 1.4. Statistical Analyses

Statistical analyses were conducted using JMP 13 software (SAS Institute Inc., Cary, NC, USA). Given the exploratory pilot design and the small sample in the EP+/PVD− cohort, a formal a priori sample-size calculation was not performed. Continuous outcomes were analyzed using nonparametric tests, which are appropriate for small samples and do not assume normality. Categorical variables were analyzed using Fisher’s exact tests as appropriate. Statistical significance was set at *p* < 0.05, and results are interpreted as hypothesis-generating.

## 2. Results

### 2.1. Demographics

Five cases were included in the EP group (FTMHs with EP without PVD) and 32 cases were included in the non-EP group (FTMHs without EP and without PVD). The mean age of the non-EP group and EP group was 66.6 ± 9.0 years and 63.4 ± 8.1 years, respectively. There was no statistical difference in the mean age of eyes in the EP group and eyes in the non-EP group (*p* = 0.25).

Both the EP group and non-EP group had 40% and 41% male patients (2 eyes and 13 eyes, respectively). The mean axial length in the EP group was 24.3 ± 1.7 mm, and that in the non-EP group was 24.8 ± 1.9 mm (*p* = 0.29). In the non-EP group, there were 9 eyes (28%) with an axial length exceeding 26 mm, whereas there was 1 eye (20%) with an axial length exceeding 26 mm in the EP group. The baseline characteristics and clinical data of the patients are shown in Table 1.

### 2.2. Clinical Characteristics and Clinical Course of FTMHs

FTMH characteristics were determined based on findings from OCT and intraoperative records. The average preoperative minimum MH size was 285.6 ± 147.4 μm in the EP group and 261.3 ± 145.6 μm in the non-EP group (*p* = 0.35). The average preoperative basal MH size was 702.6 ± 397.4 μm in the EP group and 546.5 ± 309.1 μm in the non-EP group (*p* = 0.16).

EP was detected in all eyes in the EP group and no eyes in the non-EP group. ERM was detected in all eyes in the EP group (100%) and nine eyes in the non-EP group (28%) (*p* = 0.004).

In the present study, all eyes received pars plana vitrectomy with ILM peeling and exchanging 25% SF6. Among the EP group, 3 eyes (60%) had FTMHs sealed. The 2 FTMH-unsealed eyes underwent second surgeries exchanging 25% SF6, resulting in FTMH sealing. On the other hand, all the FTMHs were sealed in the non-EP group after the first surgery. There was a significant difference in the closure rate between the EP group and non-EP group (*p* = 0.015). Additionally, when the non-EP group was divided and compared based on the presence or absence of ERM, there was no significant difference in the initial hole closure rate (*p* = 1.00).

The percentage of EZ defects after MH closure was 60% in the EP group and 6.3% in the non-EP group at 6 months postoperatively. There was thus a significant difference between the EP and non-EP groups (*p* = 0.01).

### 2.3. Visual Acuity

**Figure 2:**
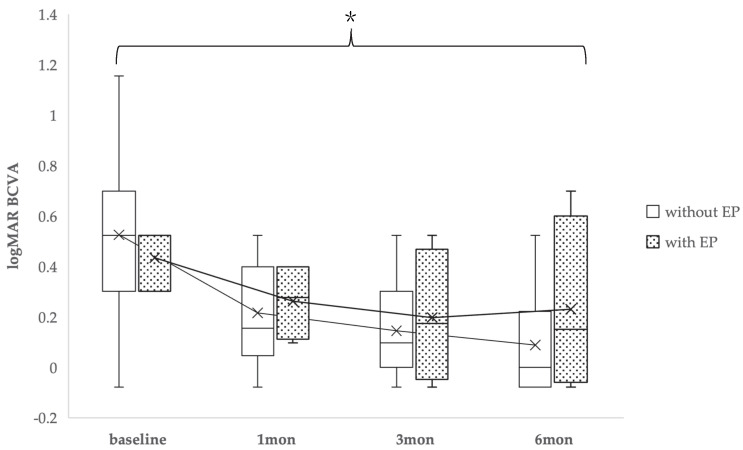
Changes in best-corrected visual acuity (logMAR BCVA) over time in patients with full-thickness macular holes (FTMHs) with and without epiretinal proliferation (EP). Box-and-whisker plots show values at baseline and at 1, 3, and 6 months postoperatively. The dotted boxes represent the EP group, and the open boxes represent the non-EP group. A significant difference in the change from baseline to 6 months was observed between the two groups (*: *p* = 0.04).

BCVA was checked before and after surgery or after sealing of the FTMH in all cases. The average preoperative logMAR BCVA and at 1, 3, and 6 months postoperatively was 0.52 ± 0.27, 0.21 ± 0.23, 0.14 ± 0.21, and 0.09 ± 0.16 in the non-EP group and 0.43 ± 0.12, 0.26 ± 0.16, 0.19 ± 0.26, and 0.23 ± 0.35 in the EP group, respectively. There were no significant differences between the two groups at each time point (preoperatively, and at 1, 3, and 6 months postoperatively). However, when comparing baseline to 6 months of logMAR BCVA, a significant difference was observed (*p* = 0.04) (Figure 2).

**Figure 3:**
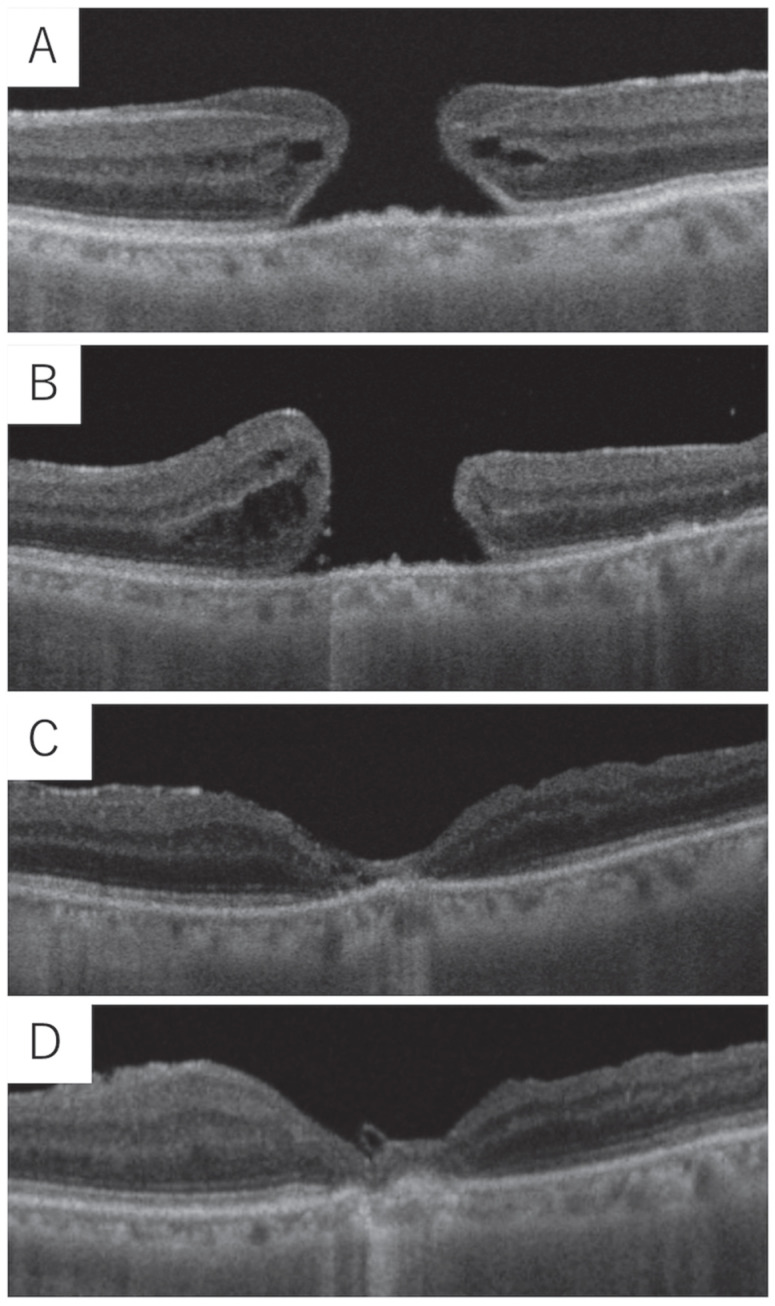
A 65-year-old male patient with an axial length of 25.35 mm and preoperative logMAR best-corrected visual acuity (BCVA) of 0.30 underwent combined cataract and vitrectomy surgery for a full-thickness macular hole (FTMH) with epiretinal proliferation (EP) and mild cataract. (**A**). Preoperative OCT showing FTMH with EP. Minimum macular hole size was 478 μm. (**B**). OCT at the first postoperative visit showing non-closure of the macular hole, leading to reoperation. (**C**). OCT 1 month postoperatively showing macular hole closure with an extensive ellipsoid zone (EZ) defect and logMAR BCVA of 0.52. (**D**). OCT 6 months postoperatively showing macular hole closure and improved foveal contour, but persistent EZ defect with logMAR BCVA of 0.69.

Figure 3 presents a case of FTMH with EP without PVD, which resulted in non-closure. In this case, an additional surgery exchanging 25% SF6 resulted in FTMH closure, but EZ loss was persistent.

**Figure 4:**
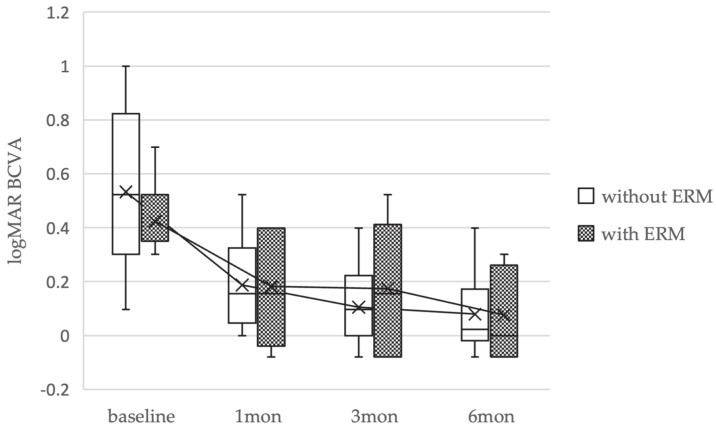
Changes in best-corrected visual acuity (BCVA) for cases of full-thickness macular holes (FTMHs)without posterior vitreous detachment treated with epiretinal membrane (ERM) or without ERM, using box-and-whisker plots.

Additional analysis was conducted of cases with ERM within the non-EP group. The baseline characteristics and clinical data of the patients are shown in Table 2. EZ defect was present in 2 eyes (22%) with ERM and no eyes (0%) without ERM 6 months postoperatively (*p* = 0.07). The average logMAR BCVA preoperatively and 1, 3, and 6 months postoperatively was 0.42 ± 0.21, 0.18 ± 0.21, 0.17 ± 0.24, and 0.07 ± 0.16 in the ERM group and 0.55 ± 0.28, 0.22 ± 0.23, 0.13 ± 0.19, and 0.09 ± 0.17 in the non-ERM group. There was no significant difference between the logMAR BCVA preoperatively and at 1, 3, and 6 months postoperatively (*p* > 0.05) (Figure 4).

## 3. Discussion

A typical FTMH is caused by the vertical traction on the macula due to PVD. EP-associated LMH may lead to the formation of FTMHs even if PVD has already occurred, due to the horizontal traction exerted by the EP or ERM [7,8,13]. In this study, we focused on FTMHs with EP without PVD. LMH is thought to develop following PVD and subsequent partial detachment of foveal tissue, potentially associated with atrophic changes in Müller cells [11]. It remains unclear whether there are additional pathways leading to the gradual loss of retinal tissue that results in LMHs. FTMHs with EP without PVD may have a different pathogenesis compared to previously reported cases as FTMH progressing from EP-associated LMH.

It has been reported that stage-2 FTMHs with EP are significantly less common than stage-4 [7]. In our study, there were 32 cases of FTMHs without EP without PVD (stage 2), and only 5 cases of FTMHs with EP without PVD, indicating a clear tendency for fewer occurrences. It has been noted that FTMHs with EP are often associated with high myopia [7] and that EP is more common in myopic eyes than in emmetropic eyes with LMH [14]. However, in our small EP group, no apparent difference was observed in the myopia between the EP group and non-EP group, although the limited sample size precludes any definitive conclusion. The earlier occurrence of PVD in myopic eyes [15] is speculated to be a reason for the higher incidence of EP in myopic LMH. However, fewer cases (20%) of myopic eyes in FTMHs with EP without PVD in our study might suggest that difference in the features and etiology compared to typical stage-2 FTMHs.

In terms of visual acuity, preoperative logMAR BCVA showed no significant difference between the two groups. However, a worsening tendency was observed in the EP group, and a statistically significant difference was noted in the subsequent changes in BCVA. This result suggests that, because the preoperative visual acuity was better in the EP group than in the non-EP group, the extent of subsequent improvement might have been inherently limited. Therefore, this significant difference may not solely reflect the effect of EP itself, but could also be influenced by the difference in baseline visual acuity. Given the small EP cohort, multivariable adjustment was not feasible and this limitation is acknowledged. To more accurately clarify the independent impact of EP, future studies with a larger sample size or analyses adjusted for baseline visual acuity will be required. This limitation should be considered when interpreting the visual outcomes of this study.

Although there was no significant difference in MH size, there were significantly more cases of EZ defects (60%) in the EP group at 6 months postoperatively. Pang et al. [2,16] found that eyes with LMH associated with EP had notably worse BCVA, extensive disruption of EZ, and reduced retinal thickness at the base of LMH compared to eyes without EP. They proposed that the disruption of the outer retinal structure contributes to Müller cell proliferation and the formation of EP in eyes with LMH. Tsai et al. found that the improvement in visual function was less in the FTMHs with ERM compared to stage-2 FTMHs without ERM [8]. Takahashi et al. reported no significant differences in BCVA at baseline, MH diameter, EZ disruption length at baseline, and post-surgical BCVA between eyes with and without EP in FTMH cases [7].

Based on previous reports, the impact of EP on visual acuity is unclear, but it is suggested that it may have a negative effect on visual function, possibly involving disruptions in the outer retinal structure. Although inconclusive, our findings raise the possibility that EP may negatively impact visual function.

However, a limitation of our study is that two cases with EP did not achieve closure after the first surgery, which might have resulted in suboptimal recovery of the outer retinal structure. Additionally, although the small sample size may have prevented significant differences, there was a tendency for larger hole diameters in FTMHs with EP, which might have contributed to the worsening tendency of visual acuity in the EP group.

In terms of closure rate, although there was no significant difference in minimum MH size or basal MH size between the two groups, both sizes tended to be larger in the FTMH with EP group. It is possible that this point may lead to a significantly lower closure rate in FTMHs with EP. It was reported that a larger minimal MH size is associated with poorer closure rates [17]. On the other hand, Takahashi et al. reported that the presence of EP does not affect the closure rate of FTMHs [7].

In the EP group, PVD did not seem to occur in the macular region because there was a posterior precortical vitreous pocket in all cases. As reported by Fukushima et al. [18], EP tissue integrates with the retinal tissue at the edge of the foveal aperture and cannot be easily separated from the retina as a typical ERM can. Thus, the traction applied to the macula during the intraoperative creation of PVD might have caused enlargement of the MH, potentially leading to a lower closure rate. While no statistical significance was reached, the observed trend toward larger MH size in the EP group might point to a potential, though uncertain, difference in features and etiology relative to typical stage-2 FTMHs.

Various methods have been proposed to increase the closure rate of MHs, such as hemitemporal ILM peeling and inverted ILM peeling [19,20]. For LMHs, surgical techniques like EP embedding have been suggested to avoid postoperative hole formation [21]. Similarly, for FTMHs with EP without PVD, alternative surgical techniques rather than standard ILM peeling might be considered. These approaches may help minimize tractional stress during surgery and improve the anatomical success rate in such challenging cases.

There was a significant difference in the closure rate between the EP group and non-EP group. Also, there were no significant differences in the initial hole closure rate in eyes with and without ERM in the non-EP group. These findings, though limited, raise the possibility that lower closure rates could be more closely related to EP than to ERM. ERM-induced FTMHs, despite their smaller size, do not exhibit significant visual improvement as stage-2 FTMHs [8]. Thus, we compared clinical characteristics and logMAR BCVA between the non-EP group with and without ERM (Table 2, Figure 4). There were no significant differences in EZ defect rates between the ERM and non-ERM groups. Similarly, there were no significant differences in logMAR BCVA between the two groups, and no significant differences were observed in other clinical characteristics. While based on limited data, the present findings raise the possibility that EP may affect visual function more adversely than ERM in cases without PVD.

## 4. Conclusions

Our preliminary observations suggest that full-thickness macular holes (FTMHs) with epiretinal proliferation (EP) without posterior vitreous detachment (PVD) tend to exhibit a lower closure rate compared with stage-2 FTMHs. These results should be interpreted cautiously, as the clinical characteristics of FTMHs with EP without PVD appear to differ from those of typical FTMHs.

Given the small sample size, the generalizability of our findings remains limited. Further prospective studies with larger cohorts are warranted to confirm these preliminary results and to elucidate the underlying pathogenesis. A deeper understanding of these differences may ultimately contribute to optimizing surgical strategies for this rare and challenging subset of macular holes.

## Figures and Tables

**Table 1:** Baseline patient characteristics.

Baseline Characteristics	FTMHs Without EP and Without PVD (Non-EP Group) (32 Eyes, 32 Patients)	FTMHs with EP Without PVD (EP Group) (5 Eyes, 5 Patients)	*p* Value
Age: mean ± SD (years)	66.6 ± 9.0	63.4 ± 8.1	0.25
Sex: n (%)			
Male	13 (41)	2 (40)	
Female	19 (59)	3 (60)	
BCVA: logMAR, mean ± SD	0.46 ± 0.20	0.32 ± 0.17	0.04
Axial length (mm, mean ± SD)	24.8 ± 1.9	24.3 ± 1.7	0.2
Combined cataract surgery: n (%)	27 (84%)	5 (100%)	
Without combined cataract surgery: n (%)	5 (16%)	0 (0%)	
Pseudophakia	5	0	
Minimum MH size ± SD (μm)	261.3 ± 145.6	285.6 ± 147.4	0.35
Basal MH size ± SD (μm)	546.5 ± 309.1	702.6 ± 397.4	0.16
ERM: n (%)	9 (28)	5 (100)	0.004

SD = Standard deviation; BCVA = best-corrected visual acuity; logMAR = logarithm of minimum angle of resolution; MH = macular hole; FTMHs = full-thickness macular holes; EP = epiretinal proliferation; PVD = posterior vitreous detachment; ERM = epiretinal membrane.

**Table 2:** Baseline characteristics of ERM cases within the non-EP group.

Baseline Characteristics	FTMHs Without ERM and Without PVD (23 Eyes, 23 Patients)	FTMHs with ERM and Without PVD (9 Eyes, 9 Patients)	*p* Value
Age: mean ± SD (years)	66.7 ± 7.6	66.2 ± 12.3	0.46
Sex: n (%)			
Male	10 (43)	3 (33)	
Female	13(57)	6 (66)	
BCVA: logMAR, mean ± SD	0.49 ± 0.20	0.37 ± 0.15	0.09
Axial length (mm, mean ± SD)	24.7 ± 1.8	24.8 ± 1.9	0.42
Combined cataract surgery: n (%)	19 (83%)	8 (88%)	
Without combined cataract surgery: n (%)	4 (17%)	1 (11%)	
Pseudophakia	4	1	
Minimum MH size ± SD (μm)	257 ± 96.1	272 ± 237.7	0.14
Basal MH size ± SD (μm)	516 ± 316.1	623.4 ± 293.4	0.14

SD = Standard deviation; BCVA = best-corrected visual acuity; logMAR = logarithm of minimum angle of resolution; MH = macular hole; FTMH = full-thickness macular hole; ERM = epiretinal membrane.

## Data Availability

The data supporting the findings of this study are available from the corresponding author upon reasonable request. However, the sharing of individual patient data is restricted due to ethical and privacy regulations approved by the Institutional Review Board of St. Marianna University School of Medicine (Approval No. 7016).
